#  “I Am but Mad North-northwest: When the Wind is Southerly I Know a Hawk from a Handsaw”

**DOI:** 10.3201/eid1401.000000

**Published:** 2008-01

**Authors:** Polyxeni Potter

**Affiliations:** * Centers for Disease Control and Prevention, Atlanta, Georgia, USA

**Keywords:** Alaska, Fred Machetanz, painting, glazing technique, art and science, Alaska Natives, Arctic, climate change, about the cover

**Figure:**
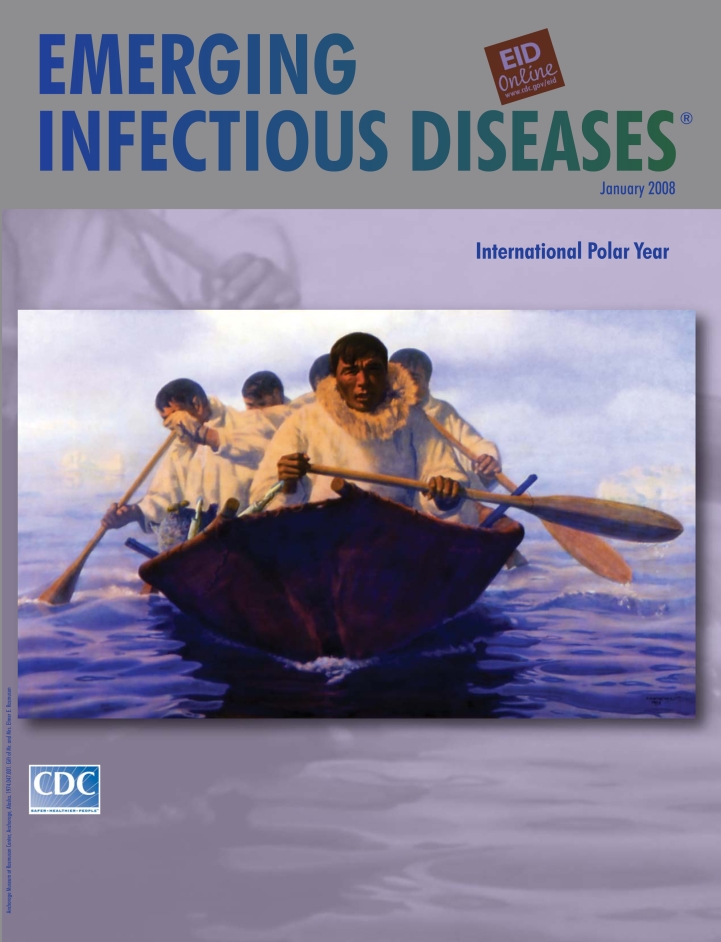
**Fred Machetanz (1908–2002). Quest for Avuk (1973).** Oil on board (81.3 cm × 130.8 cm). Anchorage Museum at Rasmuson Center, Anchorage, Alaska. 1974.047.001. Gift of Mr. and Mrs. Elmer E. Rasmuson

──William Shakespeare, Hamlet

“The true north strong and choked with ice,” wrote Canadian poet Al Purdy, about the Arctic (*1*). “The sea ... was like the concentrated essence of all the blue that ever was; I could feel that blue seep into me and all my innards change colour. And the icebergs! They were shimmery lace and white brocade, and they became my standard for the word *beauty*” (*2*). Purdy’s thrill at drifting with “the tides on Cumberland Sound and its blue fiord, [where] bergs and growlers are always in sight, even at the height of summer,” (*1*) echoes the experience of many who visit the North; among them, Fred Machetanz, painter of iconic Alaska.

A native of Kenton, Ohio, educated at Ohio State University, the American Academy of Art in Chicago, and the Arts Students League in New York, he ventured to Unalakleet, a tiny fishing village on the Bering Sea, in 1935 to visit his uncle. Captivated by the landscape, he moved there to celebrate it in his work for years to come. “I was just nuts about Alaska” (*3*).

Artists have long traveled to the icy North. During the 18th and 19th centuries, as part of explorations, they documented discoveries and sought adventure and new cultures. John Webber, official painter for Captain James Cook’s voyage (1776–1780) produced countless expertly painted records. By the end of European exploration and after the purchase of Alaska in 1867 from the Russians, travel increased. Naturalists and conservationists, John Burroughs, John Muir, and others, reported on their visits to the glaciers. North American artists, among them Eustace Ziegler, Ted Lambert, Sidney Laurence, Jules Dahlager, migrated to paint their romantic vision of “the last frontier” with its pristine wilderness and sparse inhabitants close to the land. Some of these visitors became the best landscape painters of the day. The art of Alaska’s own populations, a long and rich tradition, was influenced by the onslaught of imported forms (*4*).

Much history of the Eskimo culture of North America in early 20th century, comes to us from the work of Danish anthropologist Knud Rasmussen, whose expedition crossed North America from east of Baffin Land to Alaska and across the Bering Straight to Siberia and lived to report conditions more inclement and dangerous for humans that nearly anywhere else in the world. “Cold and mosquitoes, / these two pests / come never together,” goes the Iglulik song, “I lay me down on the ice, / Lay me down on the snow and the ice, / Till my teeth fall chattering” (*5*).

The relationship between humans and the physical world is widely explored in Inuit poetry. “We fear the weather spirit of earth, which we must fight against to wrest our food from land and sea. We fear Sila [the weather]” (*6*). Locals and sourdoughs of a bygone era under these extreme conditions, their rules for life and survival from snowstorm to snowstorm, the majestic surroundings and wildlife became Machetanz’ work; during the early years, in books, photographs, movies, and lectures; then exclusively in paintings. He set up his easel opposite the windows of his tiny cabin near Palmer and started to recreate the surroundings. Over seven decades, he exhibited widely, built a reputation, and became one of Alaska’s most beloved artists. “If anyone viewing my work has felt the beauty, the thrills and the fascination I have known in Alaska, then I have succeeded in what I set out to do” (*3*).

Though close to the artists of Alaska’s romantic era, Machetanz lived the life he painted. He embraced the wilderness, “Why that land that they want back there ain’t fit for nobody but goats, writers and artists” was the official opinion on the space staked out for his cabin (*3*). He joined a whaling crew, paddled his own umiak, drove dog sleds. If he painted an Athabascan woman with a birch bark baby carrier, he commissioned a carrier. “That’s why we have these beautiful artifacts we’ve collected, which are made to scale, and made by experts, the natives who know them.” He could “take a model and rotate it in the sunlight and get the light and shade on it” (*3*).

The art editor of Scribner’s, once joked about a Machetanz painting, “You’ve put a cherry colored head on that Eskimo.” The painter corrected him, “If you see an Eskimo under a golden pink sun, you’re going to see a red exactly like that….People don’t realize the colors that we get here. And then we have a longer chance to look at those colors” because of the long hours of daylight in the summer and late spring (*3*).

As a young man, Machetanz visited Maxfield Parrish, then probably the most famous American artist; “…hardly a home in America existed that didn’t have a Maxfield Parrish print” (*7*). He drove to Cornish, New Hampshire, to meet him, and they became friends for life. “I have always admired the art of Maxfield Parrish and a lot of the early painters of the Renaissance ... Vermeer and Titian and those. They used a technique…where they first, on the canvas or board ... painted the entire painting in one color─white ... then ... layers of transparent color, which you could look through and eventually get the final result. It’s like putting a blue glass and a red glass over a white surface, and you could look through the blue and the red and you could see a purple, but it would be a transparent purple and quite different from an opaque purple of pigment” (*3*). This laborious technique is credited for the chill northern intensity of Machetanz’ paintings, “... each layer has to be dry before I put on another layer, and my paintings contain six to eight layers of paint and varnish, and the only way I could dry them was by the sun or the stove.” (*3*).

Quest for Avuk on this month’s cover captures a theme of everyday life. Eskimo men paddle an umiak, a lightweight skin boat of the Arctic, searching for Avuk, likely a walrus (ayvuq [Central Siberian Yupik], aiviq [Inupiaq]). The men in camouflage kuspuks of cotton canvas over their parkas wear a look of intense concentration. A rifle, a toggling harpoon, and a sealskin float are visible from the side. The lithe vessel gliding noiselessly on the frigid waters allows immediate access to the hunt beneath the surface: seals, walruses, whales; in back, ice always in the invisible horizon.

“When I get home / With a catch that does not suffice, / I usually say / It was the fish / That failed― / Up the stream” (*6*). A hard stormy winter, when the caribou left and the seals were hard to find, could spell starvation for Machetanz’ subjects, early Eskimo communities, isolated, completely dependent on traditional sources of sustenance, lashed by weather. “Life is so with us that we are never surprised…that someone has starved to death. We are so used to it ... They cannot help it, it is not their fault, it is either sila [the weather] or persaq [blizzard] or to’nraq [evil spirit, i.e., sickness]” (*6*).

“I have only my song, / Though it too is slipping from me.” Arctic populations in the United States and Canada now live largely in settled communities no longer completely dependent on walrus and fish. Long adapted to isolation and affected by infections linked to climate and culture, they are now also vulnerable to emerging plagues (*8*). Back in the 1980s, in his “Trees at the Arctic Circle,” Al Purdy contemplated the strength of these trees: “And you know it occurs to me / about 2 feet under / those roots must touch permafrost / ice that remains ice forever / and they use it for their nourishment / use death to remain alive.” Now permafrost is melting. Heavily geared for ice, Arctic populations are facing yet another bout of rough weather, a warming trend. And unlike Shakespeare’s hero, they have no need to feign madness.
